# Analysis of Bacterial Stent Colonization: The Role of Urine and Device Microbiological Cultures

**DOI:** 10.3390/antibiotics12101512

**Published:** 2023-10-04

**Authors:** Gabriele Tulone, Angela Costanzo, Nicola Pavan, Rosa Giaimo, Francesco Claps, Teresa Maria Assunta Fasciana, Anna Giammanco, Riccardo Bartoletti, Alchiede Simonato

**Affiliations:** 1 Urology Section, Department of Surgical, Oncological and Stomatological Sciences, University of Palermo, 90100 Palermo, Italy; gabriele.tulone@policlinico.pa.it (G.T.); angela.costanzo@you.unipa.it (A.C.); rosa.giaimo@policlinico.pa.it (R.G.); alchiede.simonato@policlinico.pa.it (A.S.); 2 Urology Clinic, Department of Medical, Surgical and Health Sciences, University of Trieste, 34127 Trieste, Italy; francesco.claps@asugi.sanita.fvg.it; 3 Department of Health Promotion, Maternal-Childhood, Internal Medicine of Excellence “G. D’Allesandro”, University of Palermo, 90127 Palermo, Italy; teresa.fasciana@unipa.it (T.M.A.F.); anna.giammanco@unipa.it (A.G.); 4 Department of Translational Research and New Technologies in Medicine and Surgery, University of Pisa, 56126 Pisa, Italy; riccardo.bartoletti@unipi.it

**Keywords:** ureteral stent, microbiological culture, urinary infection

## Abstract

In this study, we explored the incidence of double J (JJ) contamination of patients who underwent an endourological procedure for urinary stones and ureteral stenosis. We developed a prospective study between January 2019 and December 2021. Ninety-seven patients, 54 male and 43 female, were enrolled. Urine culture was taken during four steps: before stent insertion, a sample from selective renal pelvis catheterization, a sample two days after the JJ insertion and finally, after the stent removal procedure. At the time of the stent removal, 1 cm of proximal and distal ends were cut off and placed in the culture for bacterial evaluation. Cohen’s kappa coefficient value (k) and concordance rates of microbiological culture results were evaluated. The study group comprised 56% of male patients. Proximal and distal stent cultures were positive in 81 and 78 patients. The concordance rate of microbiological cultures between proximal and distal double J stent is 88% (k 0.6). The most common pathogens isolated from urine and stent cultures were *Enterococcus* spp. in 52 cases and *Klebsiella* spp. in 27 cases.

## 1. Introduction

Ureteral Double–J stent placement is one of the most common urologic procedures. It is mainly used to facilitate upper urinary tract drainage when it is obstructed by urolithiasis, stricture, or malignancy or after ureteral endoscopic procedures such as transurethral lithotripsy (URL) and retrograde intrarenal surgery (RIRS) for treatment of ureteral and renal stones or ureteral trauma. The procedure is generally safe and easy, but a lot of early complications are possible, such as pain, hematuria, and dysuria [1,2], as well as long-term complications, such as stent dislocations and stent encrustation [3,4], and even more severe conditions like sepsis [5]. However, the most frequent complications of retained JJ stents are urinary tract infections (UTIs) [6], which occur most frequently if the stent is in place for a long period, as it is in chronic treatment, if it is forgotten [7] or because of the presence of ureteral lithiasis, which constitutes a frequent source of infection. Generally, UTIs are clinically characterized by recurrent dysuria, less frequent and purulent urine emission, and sepsis, but in some cases, simple bacteriuria may not be associated with clinical evidence. Removing or substituting a ureteral stent after a short period is generally recommended [8]. In the literature, several clinical trials have revealed the high frequency of bacterial colonization of indwelling ureteral stents and its correlation with the time the stent is in place [9]. Nevertheless, prophylactic antibiotic treatment is generally empirical or based on urine culture and its antibiogram [10]. The purpose of this study is to explore the risk of positive urine culture in patients with a ureteral stent in situ and the correlation rate of microbiological culture between urine culture and stent culture to investigate the potential utility of stent culture in the management of prophylactic or curative antibiotic therapy, to reduce the risk of potential proliferation and possible hematogenous dissemination of bacteria. 

## 2. Results

The study group comprised 97 patients. The median age was 60 (IQR 50–67). Of these, 56% were male and 44% female. Regarding comorbidities, 80% of patients had diabetes mellitus and 88% suffered from chronic kidney disease. The Charlson Comorbidity Index (CCI) was 0 for 13%, 1 for 14%, and ≥2 for 70% of patients; 59% of them had a history of stone-related surgery (Table 1). The median indwelling time of the ureteral stent was 60 (IQR 29–88) days. Only 3% of patients had a positive urine culture before the double J catheter insertion, 32% had a positive urine culture while it was in situ, and 11% developed a positive urine culture at the removal. Ureteral stents are more frequently the site of bacterial colonization than urine cultures taken from indwelling catheters and selective renal urine (82.5% and 78.4%, respectively, for proximal and distal doble J vs. 33% and 21.6%, T2 and T3 respectively). Our study analyzed the proximal and distal portions of ureteral stents removed after endoscopic procedures. We verified that proximal stent culture was positive in 82% of cases and distal stent culture in 78% of cases. Selective renal pelvis urine cultures of a sample collected during surgery were positive in 22% of patients. The analysis shows a prevalence of mixed flora in the cultures rather than a single microorganism. 42.5% and 36.8% of mixed flora were reported in the proximal and distal JJ cultures, respectively. The analyses carried out show that the number of contamination is equal to 80%. The selective renal urine cultures demonstrated a high incidence of *Enterococcus* spp. (23.8%) and *Klebsiella* spp. (28.6.%). Similarly, for stent culture, *Enterococcus* spp. has an incidence of 20% for proximal J and 15.8% for distal J and has been found *Klebsiella* spp. in 6.3% and 9.2% respectively for proximal and distal J culture (Table 2). In addition to investigating the incidence of positive cultures in the analyzed samples and the characterization of the most frequently encountered species, we also attempted to evaluate the concordance of the cultures among the analyzed samples. The reason is to evaluate whether it is sufficient to perform a single culture, for example, from an intermediate stream, or if it is necessary to analyse multiple samples, in our case from devices and selective urine from the kidney, due to the risk of having more pathogens than otherwise would not be recognized except in a late phase of infection such as in the course of bacteremia. The concordance rate between microbiological cultures of proximal and distal JJ stents is 88% (k 0.6). There is low concordance between the microbiological results of the T2 sample and the proximal and distal ureteral stent, 41.3% and 48.3% respectively (k 0.02 and 0.11). Low concordance was also detected between the T3 sample and proximal and distal stent culture, 50% and 56.1%, respectively (k 0.13 and 0.22). The Concordance between the T4 urine culture and double-J cultures was 35.2% for the proximal and 40.7% for the distal stent (k 0.06 and 0.09) (Table 3). The rate of complications was very low: 8.2% and 4.1%, respectively, for UTIs and sepsis (Table 1). All patients enrolled in the study were contacted by telephone to check for any infectious complications. In particular, the onset of fever, hematuria or re-hospitalization was assessed.

## 3. Discussion

Ureteral stents are medical devices commonly used in routine urology practice. This study evaluates the incidence of ureteral stent colonization and the concordance rate of microbiological results. There are studies in the literature that aimed to research the incidence of bacteriuria and bacterial colonization in JJ stents positioned for urinary pathologies [11,12]. Shabeena et al. report a high incidence of bacterial colonization in the devices and a correlation with the duration of stent retention. Riedl et al. instead find bacterial colonization in 100% of patients with permanent stents and enterococcus as the main colonizing agent.

This work is the first study in the literature that evaluates the concordance between different parts of the ureteral stent and with the classic urinary cultures. 77% of the patients underwent lithotripsy surgery, which led to the placement of the ureteral stent to prevent stricture formation and post-operative flank pain. The remainder underwent periodic replacement of the ureteral stents for obstructive ureteral pathologies. All patients received empiric antibiotic therapy according to European guidelines unless there was a positive urine culture, in which case targeted therapy was performed. Our findings show that not all selective renal urine cultures are positive at the second procedure, although there is a positive proximal and distal double culture. In the literature, there are many studies assessing the risk of colonization of urethral stents, and the results are conflicting. Klis et al. found bacteria in all stents [13], and Kehinde et al. in nearly 42% [9]. Our research shows a high concordance rate between proximal and distal double cultures, 88%, as expected. An unexpected finding was the low concordance rate between stent cultures and selective kidney urine, 41% with the proximal curl and 48.3% with the distal curl, regardless of how long the stent had been in place. This is different to other studies. Riedl et al. noted a 45% risk of urine infection during short–term stenting and 100% during long–term stenting [12]. This discovery represents an important fact for the urologist in the management of patients with ureteral stents. In clinical practice, a complication of the placement and presence over time of a stent is the onset of UTI, with an incidence of 11% reported in the literature [5]. A standard strategy for stent-associated UTI prevention is the removal of these implants as soon as possible. In the Vallee review, it is demonstrated that although the conventional treatment of urinary tract infections has been using antibiotics, bacteria can still survive on indwelling medical devices and patients are at risk of UTIs, including complications like sepsis in 15% of cases [14]. This potentially lethal complication can be caused by several factors, such as the ability of indwelling stents to cause the vesicoureteral reflux of urine from the bladder into the renal collecting system [15]. Other studies demonstrate that indwelling stents decrease ureteral peristalsis [16]. Bacteria can enter the renal parenchyma and access the renal circulatory system, leading to bacteremia, the first stage of urosepsis. The question is whether the risk of urosepsis during antibiotic therapy is due to the resistance of bacteria in the biofilm to antibiotics or perhaps to a non-specific antibiotic therapy against the infecting microorganism. As a result, other strategies need to be considered. Developments have been made in biomaterial technology, with promising results [17,18], but this is not enough. Our study demonstrates the need to routinely perform a culture examination of the proximal and distal double J ureteral stent, selective urine cultures of the kidney and standard urine cultures from bladder urine samples, and the effectiveness of this course of action. Given the low concordance between selective urine and ureteral stents, it is not clear from our study if there is a possible relation between contamination of JJ and an infective complication.

Moreover, our data confirm the modification of the etiological agents with which we have to confront ourselves for UTI. Until a few decades ago, the most frequent etiological agent was Escherichia coli. Today, more aggressive microorganisms are widespread and have a greater incidence of resistance. These include, for example, *Klebsiella* spp. which in our study has the highest incidence in samples of kidney selective urine and equal incidence to E. Faecalis in the BUC in stay double J. These data confirm the World Health Organization’s evaluation of the antimicrobial global resistance report on surveillance [19]. The analyses found the presence of enterococcus in 23% of selective urine cultures despite prophylaxis with fifth-generation cephalosporins. This shows that reflection is necessary on a possible change in the empirical therapy administered to the patient population being tested.

The limitations of our study are the low number of patients, the number of cases of urosepsis, only 4/97 patients, and the evaluation of comorbidities like diabetes as risk factors for stent colonization. We did not evaluate any correlation between gender and antibiotic exposure because several studies have not demonstrated any correlation with stent colonization [20]. Another study limitation is that several cultures were performed even during antibiotic therapy. This certainly made the choice of antibiotics difficult. Further studies will be necessary to verify a concordance between septic patients’ blood and urine cultures and devices.

## 4. Materials and Methods

A prospective study was performed from January 2019 to December 2021. We collected data from patients who underwent a Double J ureteral stent procedure in the Urological clinic center of Palermo, Paolo Giaccone Hospital. We included all consecutive patients in the period of the study. Patients underwent ureteral stenting in 23% of cases for ab extrinsic stenosis (caused by any process that lies outside the ureter and causes a narrowing of the ureter, such as a tumor or retroperitoneal fibrosis). The other patients have been treated for nephro or ureteral lithiasis. This group includes patients with previous emergency stent placement for complicated UTIs or hydronephrosis. An elective subsequent treatment for lithiasis was performed with ureteral lithotripsy (ULT), retrograde intrarenal surgery (RIRS) or percutaneous nephrolithotripsy (PCNL) based on stone localization and size. All patients treated in an urgent setting without a urine analysis before the procedure received antibiotic prophylaxis with a fifth-generation cephalosporin 30 min before surgery, which was prolonged till the fifth postoperative day. Patients treated electively received antibiotic prophylaxis according to European guidelines [21]. Patients with cardiac valve prostheses were treated according to recent cardiological guidelines [22]. The primary endpoint was to report the incidence of stent colonization and to explore a possible concordance between urine and stent pathogens. The study protocol was approved by the local ethics committee. All the participants gave informed written consent after a complete study description. All the clinical data were recorded,, and the patients were monitored for 30 days after surgery to collect peri and post-operative complications.

### 4.1. Microbiology Laboratory Technique

The urine culture was performed during four steps: before stent insertion using a midstream sample (T1) at the hospitalization without any device in stay and a bladder sample just before the treatment with the JJ in stay (T2). A selective renal pelvis sample was collected during surgery with a sterile ureteral catheter after double J removal (T3) and a urine sample from midstream urine after the stent removal procedure (T4). In addition, 1 cm of proximal and distal stent ends were cut off and placed in a culture for bacterial growing evaluation.

### 4.2. Microbiological Analysis

#### 4.2.1. Urine Culture Using the Calibrated Loop/Surface Streak Method

The plate count method (spread plate technique) was used for the isolation and quantification of the microorganisms. Briefly, the urine samples were mixed well by shaking in sterile bottles. Using a sterile loop of 1 µL was transferred onto the surface on 5% (*v*/*v*) sheep blood agar, MacConkey agar and Sabouraud agar (Becton Dickinson, Franklin Lakes, NJ, USA) [23]. Using a sterile L rod spreader, the samples were spread evenly on the surface of each nutrient agar. The plates were incubated at 37 °C in an aerobic atmosphere overnight. After incubation, the number of colonies in each plate was counted. As a cutoff for UTI, was counted only the morphology of the colony grown with several colonies of ≥100 (number of bacteria is ≥10^5^ cfu/mL) [24,25]. A bacteriuria threshold ≥ 10^5^ CFU/mL was chosen, as recommended in the presence of an endourological catheter.

#### 4.2.2. Stent Culture

The prepared stent was put into a sterile container (Bandelin, Germany) and completely covered with 5–10 mL of Brain Heart Infusion Broth (Becton Dickinson, Franklin Lakes, NJ, USA). To disrupt the advice microorganism on the inner surface of the stent, the specimen was vortexed for 30 s and subsequently exposed to low-frequency (40 kHz) ultrasound for 15 min [26]. Thereafter, the container was vortexed again for 30 s. Aliquots of the sonication fluid were cultivated on conventional solid media, 5% (*v*/*v*) sheep blood agar, MacConkey agar and Sabouraud agar (Becton Dickinson). The plates were incubated at 37 °C in an aerobic atmosphere overnight. Matrix-assisted laser desorption/ionization-time of flight (MALDI-TOF) mass spectrometer (Bruker Daltonics, Billerica, MA, USA was used for genus and species of microrganism [27].

### 4.3. Statistical Analysis

Descriptive analysis included frequencies and proportions for categorical variables. Medians and interquartile range (IQR) were reported for continuous ones. Variables collected included age, gender, presence of Diabetes Mellitus or chronic kidney disease (CKD), operative side, history of stone-related surgeries and time of double-J in-stay. We expressed the degree of agreement among different microbiological cultures’ combinations. Particularly, the degree of agreement among bladder urinary culture either during in-stay double-J stent or after its removal, renal pelvis (selective) urinary culture, distal and proximal double-J cultures was evaluated concordance (ranging from 0% to 100%), which was defined as a confirmatory result between the cultures investigated. Usually, concordance between two rates is generally expressed according to Cohen’s kappa coefficient value, ranging from −1.00 to 1.00 [28]. According to Cohen’s kappa coefficient, concordance was classified as a slight concordance for coefficient values of 0.01–0.20, fair concordance for 0.21–0.40, moderate concordance for 0.41–0.60, good concordance for 0.61–0.80, and very good concordance for 0.81–1.00. A further sensitivity descriptive analysis focusing on the bacterial flora identified was performed. No agreement was achieved for negative coefficient values. Statistical analyses were performed using R v.3.6.3 (R-Foundation for Statistical-Computing).

## 5. Conclusions

Our study demonstrates that stents are more frequently colonized by bacteria than mid-stream urine and selective kidney urine. Furthermore, there is often agreement in culture between the proximal and the distal curl of the ureteral stent. Despite the low presence of infectious complications, performing a double J stent culture may be useful to reduce cases of empirical treatment. Further study will be necessary to confirm if an infective post-operative complications can be related to a contamination of double j.

## Figures and Tables

**Table 1:** Descriptive clinical features of the patients included in the current analysis.

Variables	
Patients, *n*. (%)	97 (100.0)
Age (years), median (IQR)	60 (50–67)
Sex, *n*. (%)	
Male	54 (55.7)
Female	43 (44.3)
DM, *n*. (%)	77 (79.4)
CKD, *n*. (%)	85 (87.6)
CCI, *n*. (%)	
0	13 (13.4)
1	14 (14.4)
≥2	70 (72.2)
Side, *n*. (%)	
Right	53 (54.6)
Left	39 (40.2)
Bilateral	5 (5.2)
History of stone-related surgery, *n*. (%)	57 (58.8)
2nd procedure, *n*. (%)	
URS/RIRS	40 (41.2)
PCNL	3 (3.1)
SWL	32 (33.0)
none	22 (22.7)
In-stay double J (days), median (IQR)	60 (29–88)
Perioperative Complications, *n*. (%)	
UTIs	8 (8.2)
Sepsis	4 (4.1)

Abbreviations are as follows: IQR: interquartile range; DM: Diabetes Mellitus; CKD: Chronic Kidney Disease; URS: Ureterorenoscopy; RIRS: Retrograde Intrarenal Surgery; PCNL: percutaneous nephrolithotripsy; SWL: Shock Wave Lithotripsy: UTIs: Urinary Tract Infections.

**Table 2:** Microbiological culture results variability among 97 patients who underwent double-J.

Variables	(T1): BUC Pre-DoubleJ ^a^	(T2): BUC In-Stay DoubleJ ^b^	Proximal-J Culture ^c^	Distal-J Culture ^d^	(T3): RP Culture ^e^	(T4): BUC Post-DoubleJ Removal ^f^
Patients with a positive culture, *n*. (%)	3 (3.1)	32 (33.0)	80 (82.5)	76 (78.4)	21 (21.6)	11 (11.3)
Bacteria, *n*. (%)						
*E. coli*	1 (33.3)	4 (12.5)	7 (8.8)	4 (5.3)	0 (0.0)	1 (9.1)
*E. faecalis*	0 (0.0)	6 (18.8)	16 (20.0)	12 (15.8)	5 (23.8)	3 (27.3)
*E. faecium*	0 (0.0)	0 (0.0)	0 (0.0)	0 (0.0)	0 (0.0)	0 (0.0)
*Klebsiella* spp.	0 (0.0)	6 (18.8)	5 (6.3)	7 (9.2)	6 (28.6)	3 (27.3)
*Staphylococcus haemoliticus*	0 (0.0)	0 (0.0)	2 (2.5)	5 (6.6)	0 (0.0)	0 (0.0)
*Staphylococcus epidermidis*	0 (0.0)	0 (0.0)	6 (7.5)	5 (6.6)	0 (0.0)	1 (9.1)
*Staphylococcus lugdunensis*	0 (0.0)	0 (0.0)	0 (0.0)	2 (2.6)	0 (0.0)	0 (0.0)
*Candida albicans*	0 (0.0)	2 (6.3)	1 (1.3)	3 (3.9)	1 (4.8)	1 (9.1)
*Candida glabrata*	0 (0.0)	1 (3.1)	0 (0.0)	0 (0.0)	1 (4.8)	0 (0.0)
*Pseudomonas aeruginosa*	0 (0.0)	2 (6.3)	5 (6.3)	5 (6.6)	1 (4.8)	0 (0.0)
*Candida kefyi*	0 (0.0)	0 (0.0)	0 (0.0)	0 (0.0)	0 (0.0)	0 (0.0)
*citrobacter*	0 (0.0)	0 (0.0)	0 (0.0)	0 (0.0)	0 (0.0)	0 (0.0)
*Acinetobacter jejuni*	0 (0.0)	0 (0.0)	0 (0.0)	0 (0.0)	0 (0.0)	0 (0.0)
*Staphylococcus capitis*	1 (33.3)	6 (18.8)	0 (0.0)	1 (1.3)	1 (4.8)	1 (9.1)
*Staphylococcus species*	0 (0.0)	0 (0.0)	2 (2.5)	0 (0.0)	0 (0.0)	0 (0.0)
*Staphylococcus hominis*	0 (0.0)	0 (0.0)	0 (0.0)	1 (1.3)	0 (0.0)	0 (0.0)
*Corynebacterium aurimucosum*	0 (0.0)	0 (0.0)	1 (1.3)	1 (1.3)	0 (0.0)	0 (0.0)
*Corynebacterium jeikeium*	0 (0.0)	0 (0.0)	0 (0.0)	1 (1.3)	0 (0.0)	0 (0.0)
*Streptococcus agalactaie*	1 (33.3)	1 (3.1)	1 (1.3)	1 (1.3)	0 (0.0)	0 (0.0)
*Morganella morganii*	0 (0.0)	1 (3.1)	0 (0.0)	0 (0.0)	0 (0.0)	0 (0.0)
Mixed flora, *n*. (%)	0 (0.0)	3 (9.4)	34 (42.5)	28 (36.8)	6 (28.8)	1 (9.1)

Abbreviations include BUC: bladder urine culture; RP: renal pelvis (selective). Notes: ^a^—Urine culture Information was not available in 35 patients, ^b^—Urine culture Information was not available in 15 patients, ^c,d^—Culture Information was available in all patients, ^e^—Urine culture Information was not available in 39 patients, ^f^—Urine culture Information was not available in 43 patients.

**Table 3:** Cohen’s kappa coefficient and concordance rates of microbiological culture results.

	Positive proximal double-J culture	Negative proximal double-J culture	
Positive BUC in-stay double-J	30	2	Cohen’s kappa coefficient value 0.13
Negative BUC in stay double-J	39	11	Concordance rate 50.0%
	Positive distal double-J culture	Negative distal double-J culture	
Positive BUC in-stay double-J	30	2	Cohen’s kappa coefficient value 0.22
Negative BUC in stay double-J	34	16	Concordance rate 56.1%
	Positive distal double-J culture	Negative distal double-J culture	
Positive proximal double-J culture	72	8	Cohen’s kappa coefficient value 0.61
Negative proximal double-J culture	4	13	Concordance rate 88.0%
	Positive proximal double-J culture	Negative proximal double-J culture	
Positive RP culture	18	3	Cohen’s kappa coefficient value 0.01
Negative RP culture	31	6	Concordance rate 41.3%
	Positive distal double-J culture	Negative distal double-J culture	
Positive RP culture	19	2	Cohen’s kappa coefficient value 0.12
Negative RP culture	28	9	Concordance rate 48.3%
	Positive BUC in-stay double-J	Negative BUC in stay double-J	
Positive RP culture	9	8	Cohen’s kappa coefficient value 0.33
Negative RP culture	6	23	Concordance rate 69.6%
	Positive proximal double-J culture	Negative proximal double-J culture	
Positive BUC post-removal double-J	10	1	Cohen’s kappa coefficient value 0.06
Negative BUC post-removal double-J	34	9	Concordance rate 35.2%
	Positive distal double-J culture	Negative distal double-J culture	
Positive BUC post-removal double-J	10	1	Cohen’s kappa coefficient value 0.09
Negative BUC post-removal double-J	31	12	Concordance rate 40.7%
	Positive BUC in-stay double-J	Negative BUC in stay double-J	
Positive BUC post-removal double-J	6	2	Cohen’s kappa coefficient value 0.19
Negative BUC post-removal double-J	17	24	Concordance rate 61.2%

Abbreviations are as follows: BUC: bladder urine culture; RP: renal pelvis (selective).

## Data Availability

Data is available upon request.
